# Clinical Factors Associated with Ventilator-Free Days in Newborns with Persistent Pulmonary Hypertension of the Newborn: A Retrospective Cohort Study in Thailand

**DOI:** 10.3390/jcm15114377

**Published:** 2026-06-05

**Authors:** Sirichan Larpnarongchai, Gunlawadee Maneenil, Anucha Thatrimontrichai, Supaporn Dissaneevate, Manapat Praditaukrit, Pattima Pakhathirathien

**Affiliations:** Division of Neonatology, Department of Pediatrics, Faculty of Medicine, Prince of Songkla University, Songkhla 90110, Thailand; muggle_name@hotmail.com (S.L.); tanucha@medicine.psu.ac.th (A.T.); dsupapor@medicine.psu.ac.th (S.D.); manapat.p@psu.ac.th (M.P.); ppattima@medicine.psu.ac.th (P.P.)

**Keywords:** neonatal intensive care, newborn, outcomes, pulmonary hypertension, respiratory distress syndrome, ventilator-free days

## Abstract

**Background/Objectives**: Ventilator-free days (VFDs) are a useful composite measure to assess both survival and duration of mechanical ventilation in critically ill patients. This study aimed to determine the factors associated with (VFDs) in neonates with persistent pulmonary hypertension of the newborn (PPHN) and to compare VFDs according to the etiology and severity of PPHN. **Methods**: We conducted a retrospective cohort study of neonates diagnosed with PPHN between 2013 and 2023. VFDs were defined as days alive and free of mechanical ventilation within the first 28 days. Severe-to-critical PPHN group was defined as an oxygenation index (OI) > 25. **Results**: Among 175 neonates, the median (interquartile range [IQR]) VFDs were 20 (9–23) days. The factors independently associated with fewer VFDs included maximum OI > 40 (adjusted hazard ratio [aHR] 3.5, 95% confidence interval [CI]: 2.49–4.9), receiving more than two inotropic drugs (aHR 2.27, 95% CI: 1.49–3.45), acute kidney injury (AKI) (aHR 1.54, 95% CI: 1.1–2.17), and ventilator-associated pneumonia (VAP) (aHR 3.42, 95% CI: 1.8–6.48). The median (IQR) number of VFDs in neonates with PPHN secondary to respiratory distress syndrome (RDS), pneumonia/sepsis, meconium aspiration syndrome, and transient tachypnea of the newborn were 16 (0–22), 17 (7–21), 22 (11–24), and 22 (15–24) d, respectively (*p* = 0.023). Neonates in the severe-to-critical group had markedly fewer VFDs than those in the mild-to-moderate group (8.5 vs. 22 d, *p* < 0.001). **Conclusions**: Infants with PPHN from RDS had the fewest VFDs. A maximum OI > 40, use of multiple inotropic agents, AKI, and VAP were associated with a low number of VFDs. Given the retrospective, single-center design, these findings are associative and hypothesis-generating, requiring prospective multi-center validation. Nonetheless, VFDs remain a comprehensive measure of both mortality and respiratory morbidity in this population.

## 1. Introduction

Persistent pulmonary hypertension of the newborn (PPHN) occurs when the transition from fetal to postnatal circulation is disrupted, leading to severe hypoxemia and a high mortality rate [1,2]. The incidence of PPHN varies and has been reported as 1.9 per 1000 live births in the USA [1]. The clinical spectrum of PPHN varies from mild cases to profound hypoxemia with cardiovascular instability that requires intensive care. The leading causes of PPHN are meconium aspiration syndrome (MAS), pneumonia, and respiratory distress syndrome (RDS) [3]. Although advanced management strategies, including surfactant therapy, pulmonary vasodilators, inhaled nitric oxide, and extracorporeal membrane oxygenation (ECMO), have been used in the treatment of patients with PPHN, mortality rates remain high in resource-limited centers, and long-term neurodevelopmental impairments, such as hearing loss and learning impairment, continue to be significant concerns for patients with severe PPHN [3,4].

Ventilator-free days (VFDs) are commonly utilized as a composite outcome metric for evaluating patients with acute RDS across both adult and pediatric groups [5,6]. The 28-day VFDs represent one of the organ failure–free outcome indicators commonly applied in critical care to assess the effectiveness of therapeutic strategies and interventions [7]. VFDs are calculated as the total number of days a patient is alive without requiring mechanical ventilation. Previous studies have reported VFDs as secondary outcomes and interpreted them alongside other outcome parameters, such as duration of mechanical ventilation, vasoactive-inotrope score, improvement of the oxygenation index (OI), and mortality rate, to determine treatment efficacy [5,8]. Other studies have reported VFDs as the primary outcome when comparing the efficacy of two interventions [9,10]. Only a few recent studies have reported VFDs in neonatal populations. Therefore, this study aimed to determine the factors associated with VFDs in neonates with PPHN and to compare VFDs according to the etiology and severity of PPHN.

## 2. Materials and Methods

### 2.1. Setting and Population

This retrospective cohort study included neonates diagnosed with PPHN who were admitted to the Neonatal Intensive Care Unit (NICU) of Songklanagarind Hospital, a major tertiary referral center in Southern Thailand, from January 2013 to December 2023. Eligible cases were identified through a review of existing medical records after institutional ethics approval had been obtained. Neonates born outside the hospital, who had chromosomal or major anomalies, congenital heart disease, or congenital diaphragmatic hernias were excluded. Ethical approval for this study was granted by the Institutional Review Board and Ethics Committee of the Faculty of Medicine, Prince of Songkla University, Thailand (REC 65-471-1-1, approval date: 23 December 2022). Because of the retrospective design of the study and the use of anonymized medical record data, the requirement for informed consent was waived by the Ethics Committee.

### 2.2. Management of PPHN

The diagnosis of PPHN was based on clinical symptoms, such as differential cyanosis or labile saturation. Echocardiography was conducted to confirm the diagnosis and exclude infants with congenital structural heart defects. Increased pulmonary pressure, along with a right-to-left or bidirectional shunt across the foramen ovale or ductus arteriosus, was detected [1]. The management of PPHN was based on the NICU protocols designed for patients with PPHN. The NICU protocol offered two ventilation management options: conventional mechanical ventilation (CMV) and high-frequency oscillatory ventilation (HFOV). Infants who failed to ventilate adequately with CMV (requiring peak inspiratory pressure ≥ 25 mmHg or experiencing refractory hypoxemia) were transitioned to HFOV [11].

In 2013, inhaled nitric oxide (iNO) became available and was used in patients with an OI greater than 20–25. ECMO therapy was introduced in 2015 and was provided to patients whose OI remained above 40 for 0.5–6 h or who experienced persistent metabolic acidosis with refractory hypotension [11]. In our unit, HFOV was used for most patients with PPHN. After extubation, the patient was considered for noninvasive ventilation (NIV) at the discretion of the attending staff. The NIV modes included nasal intermittent positive pressure ventilation, nasal continuous positive airway pressure, and nasal high-frequency oscillatory ventilation. The interfaces were facemasks or nasal cannulas.

### 2.3. Data Collection

Data were obtained from the hospital’s electronic medical records system, including maternal and neonatal records. Maternal data included antenatal steroid use and delivery mode. Neonatal data included gestational age, sex, birth weight, Apgar score, timing of intubation and extubation, causes of PPHN, initial OI level (calculated when PPHN was diagnosed), maximum OI, inotropic drugs used, and details regarding iNO therapy. Documented neonatal comorbidities were pneumothorax, bronchopulmonary dysplasia (BPD), acute kidney injury (AKI), and ventilator-associated pneumonia (VAP). Clinical outcomes assessed included duration of mechanical ventilation, VFDs, length of hospitalization, and mortality.

### 2.4. Definitions

The OI was determined using the conventional formula: FiO_2_ × mean airway pressure × 100/PaO_2_. PPHN severity was evaluated according to OI values and categorized as mild (OI ≤ 15), moderate (OI 16–25), severe (OI 26–40), and very severe or critical (OI > 40) [12]. For binary comparisons, an OI > 25 was used to define the combined severe-to-critical group. We determined the severity of PPHN at the time of diagnosis.

VFDs at 28 d were defined as the number of days alive without requiring mechanical ventilation for at least 48 consecutive hours [13]. Days on noninvasive ventilation were not included. The 28 d timeframe was selected as most patients with acute lung injury would have either been successfully weaned from mechanical ventilation or not survived by that time [6,7]. The day of intubation was used as day 0. VFDs were computed as “28 minus the total number of days on mechanical ventilation,” and recorded as “0” if the infant either died within 28 days of intubation or required ventilation beyond 28 days. If a patient was extubated on day 5 and remained alive without requiring mechanical ventilation for the rest of the 28-day period, VFDs were 23. For cases involving reintubation, any period of extubation lasting less than 48 consecutive hours was not counted toward VFDs. If an infant was successfully extubated for more than 48 h but required reintubation later within the 28-day timeframe, VFDs were still calculated based on the cumulative total days on mechanical ventilation. For example, if an infant was ventilated for 5 days, successfully extubated for 5 days, and then reintubated for another 5 days before final successful weaning, the total number of days on ventilation was 10, resulting in a VFDs score of 18.

BPD was diagnosed according to the National Institute of Child Health and Human Development (NICHD) criteria [14]. VAP was diagnosed based on the Centers for Disease Control and Prevention (CDC) and National Healthcare Safety Network (NHSN) guidelines for infants under 1 year of age [15]. AKI was diagnosed according to the Kidney Disease Improving Global Outcomes (KDIGO) guidelines [16]. Diagnostic criteria included: (1) a rise in serum creatinine ≥ 0.3 mg/dL within 48 h; (2) increase in serum creatinine to ≥1.5 times baseline within the previous 7 days (known or presumed); and/or (3) urine output < 0.5 mL/kg/h for at least 6 h.

### 2.5. Statistical Analysis

Statistical analyses were performed using the Epicalc package in R (version 4.2.1; R Foundation for Statistical Computing, Vienna, Austria). Categorical data were summarized as percentages and analyzed using Fisher’s exact test or the chi-square test. The Shapiro–Wilk test was performed to assess whether the data followed a normal distribution. Continuous data were presented as mean ± standard deviation (SD) or median (interquartile range, IQR) and compared with Student’s *t*-test or Wilcoxon rank-sum test. The Kaplan–Meier method with log-rank testing and Cox proportional hazards models were applied to evaluate the cumulative probability and clinical predictors of time-to-event outcomes, including overall survival and the clinical progression of VFDs.

Variables with a *p*-value < 0.2 in the univariate analysis were subsequently included in a multivariable model using backward stepwise selection. To prevent model overfitting, the number of candidate variables was restricted to ensure an events-per-variable (EPV) ratio well above the recommended threshold of 10. Additionally, multicollinearity among the multivariable predictors was rigorously assessed using the Variance Inflation Factor (VIF), with a VIF value of <5 considered acceptable to maintain model stability. A *p*-value < 0.05 was considered statistically significant.

To address potential biases arising from the non-standard, composite nature of VFDs, where both early mortality and prolonged mechanical ventilation are assigned a value of zero, a sensitivity analysis was performed. Specifically, we conducted a restricted time-to-event analysis by excluding patients who died within 28 days (*n* = 25) and those with prolonged ventilation beyond 28 days (*n* = 8) to evaluate the true distribution of time-to-successful extubation among the remaining cohort (*n* = 142). Kaplan–Meier cumulative probability curves were reconstructed for this subgroup to ensure that the core findings were not artifactually driven by zero-inflation from the mortality and prolonged ventilation cohorts.

## 3. Results

A total of 266 neonates were diagnosed with PPHN during the study period, of whom 175 met the inclusion criteria. The 91 exclusions were due to being outborn (*n* = 65), congenital heart disease (*n* = 9), chromosomal or major congenital anomalies (*n* = 5), or congenital diaphragmatic hernia (*n* = 12). The causes of PPHN included MAS, transient tachypnea of the newborn (TTN), and congenital pneumonia/sepsis. Echocardiography confirmed the diagnosis in 172 (98.3%) infants.

Among the study population, 72 infants (41.1%) had mild PPHN, 45 (25.7%) had moderate disease, 37 (21.1%) had severe disease, and 21 (12.0%) had critically severe disease. The overall median (IQR) maximum OI was 31.6 (18.1–54.2). Three patients underwent ECMO therapy, all of whom survived.

The baseline characteristics and outcomes of neonates with PPHN, stratified by severity: mild-to-moderate (OI ≤ 25) versus severe-to-critical (OI > 25), are presented in Table 1. The severe-to-critical PPHN group had fewer VFDs, a longer duration of mechanical ventilation, and higher rates of pneumothorax and mortality than the mild-to-moderate group.

**Figure 1:**
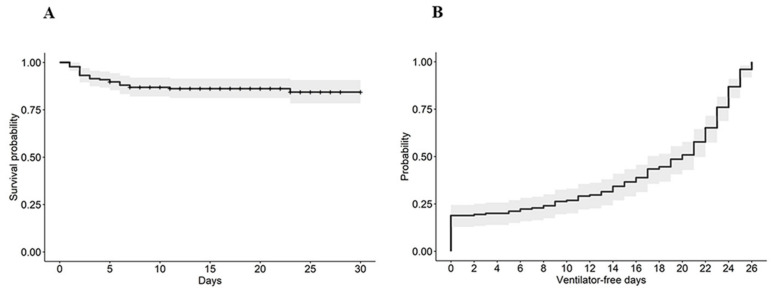
Kaplan–Meier curves of clinical outcomes in neonates with PPHN (*n* = 175). (**A**) Overall survival up to 30 days of life. (**B**) Ventilator-free days (VFDs) within 28 days of life. VFDs were defined as the number of days alive and free from mechanical ventilation during the first 28 days; infants who died within 28 days or required mechanical ventilation beyond this period were assigned a value of zero.

The overall in-hospital survival rate was 84.6% (148/175), and the 30-day cumulative survival was 84.3% (95% CI: 78.4–90.7) (Figure 1A). In contrast, VFDs at 28 days showed a wide distribution, with 18.9% (95% CI: 12.9–24.5), 22.9% (95% CI: 16.4–28.8), and 34.3% (95% CI: 26.9–40.9) of infants having VFDs of 0, 7, and 14, respectively (Figure 1B).

In a sensitivity analysis restricting the cohort to patients who achieved successful extubation within 28 days (*n* = 142), the cumulative probability curve of time-to-successful extubation remained consistent without the zero-inflation artifact (Supplementary Figure S1).

VFDs differed significantly between the mild-to-moderate and severe-to-critical PPHN groups (*p* < 0.001). The proportions of patients with VFDs = 0 in severe-to critical and mild-to-moderate groups were 40% (95% CI: 26.2–51.2) and 7.9% (95% CI: 2.8–12.7), respectively.

Of the 175 infants, 85 (48.5%) received iNO therapy, with a median (IQR) duration of 52 (24–80) h. Reflecting a patient population with higher baseline clinical severity, the median (IQR) number of VFDs were significantly lower in the iNO group than in the non-iNO group [16 (2–22) days vs. 23 (15–24) days, *p* < 0.001], and the median (IQR) duration of mechanical ventilation was significantly longer in the iNO group [7 (4–12) days vs. 5 (3–9) days, *p* = 0.034]. However, mortality did not differ significantly between the two groups (21.2% vs. 10%, *p* = 0.066).

Factors associated with VFDs in neonates with PPHN are shown in Table 2. Multivariable analysis revealed that a maximum OI ≥ 40, the use of more than two inotropic agents, and the presence of AKI or VAP were significantly associated with a lower probability of successful extubation (i.e., fewer VFDs). Forty-seven (26.9%) neonates were diagnosed with AKI. The median (IQR) VFDs were significantly lower in neonates with AKI than in those without AKI [9 (0–22) days vs. 21 (15–24) days, *p* < 0.001]. Mortality was also higher in the AKI group (36.2% vs. 7.8%, *p* < 0.001), whereas the duration of mechanical ventilation and length of hospital stay did not differ significantly between groups. In the final multivariable Cox proportional hazards model, a total of 142 successful extubation events were analyzed against 4 final covariates, yielding a robust EPV ratio of 35.5. Standard diagnostic testing demonstrated excellent model stability with no evidence of significant multicollinearity, as all retained covariates exhibited VIF below 1.10 (ranging from 1.02 to 1.10).

Among the 175 neonates, 12 (6.8%) were diagnosed with VAP. Infants with VAP had significantly fewer median (IQR) VFDs compared with those without VAP [9 (2.2–11.8) days vs. 21 (11–24) days, *p* = 0.001]. In addition, both the duration of mechanical ventilation and length of hospital stay were significantly longer in the VAP group.

A comparison of neonatal outcomes according to the underlying causes of PPHN is presented in Table 3. There were significant differences in VFDs, the duration of mechanical ventilation, length of hospital stay, and mortality among the groups. Infants with PPHN secondary to RDS had the fewest VFDs, whereas those with MAS and TTN had the most. However, the duration of mechanical ventilation was similar across the RDS, MAS, and TTN groups. Infants with PPHN secondary to pneumonia/sepsis and birth asphyxia had similar numbers of VFDs; however, the duration of mechanical ventilation was shorter in the pneumonia/sepsis group than in the birth asphyxia group.

## 4. Discussion

The number of VFDs is a commonly reported outcome in pediatric acute RDS studies [5]. In this study, the median VFDs in neonates with PPHN during the first 28 days were 20 d. Infants with PPHN secondary to RDS had the fewest VFDs and highest mortality, whereas those with TTN and MAS had comparable VFDs and the duration of mechanical ventilation but different mortality rates. In addition, severe-to critical group was associated with markedly fewer VFDs than mild-to-moderate group in infants with PPHN.

Although overall survival was relatively high, VFDs demonstrated substantial heterogeneity in respiratory outcomes among survivors. This discrepancy between relatively preserved survival and variable VFDs suggests that survival alone may underestimate disease burden in PPHN. In contrast, as a composite outcome incorporating both mortality and duration of mechanical ventilation, VFDs provide a more comprehensive clinical picture by capturing differences in respiratory recovery and illness severity. Notably, the observed differences in VFDs despite similar mortality rates across some groups further support their value in identifying clinically meaningful differences in morbidity that are not reflected by survival alone.

In addition, the distribution of VFDs showed clustering at specific values, which may reflect common clinical trajectories, including early mortality, prolonged mechanical ventilation, or successful early weaning. This pattern underscores the ability of VFDs to capture distinct pathways of disease progression and recovery in neonates with PPHN.

RDS, caused by surfactant deficiency, leads to atelectasis and hypoxemia [17,18]. Infants with RDS complicated by PPHN often require advanced respiratory support, while options such as iNO and ECMO may be limited in extremely preterm infants. Greater RDS severity has been associated with worsening PPHN [19]. Consequently, neonates with PPHN secondary to RDS tend to have fewer VFDs and exhibit higher mortality rates compared with those whose PPHN arises from other causes.

We identified a maximum OI > 40, use of more than two inotropic agents, AKI, and VAP as factors associated with fewer VFDs. OI is a well-established marker of hypoxemic severity, with values > 40 associated with the need for aggressive support and increased mortality [2,20,21,22]. Similarly, the requirement for multiple inotropic agents reflects significant hemodynamic compromise, which may prolong respiratory support [2].

AKI in PPHN may result from hypoxic injury and has been linked to worse respiratory outcomes and increased mortality [23,24]. Similarly, VAP is associated with prolonged mechanical ventilation and fewer VFDs, likely due to inflammatory lung injury and increased risk of complications such as BPD [25,26].

In this study, infants who received iNO had fewer VFDs and longer durations of mechanical ventilation, while mortality was not significantly different. This association is likely influenced by confounding by indication, as iNO is typically administered to more severely ill infants. Therefore, these findings should not be interpreted as evidence of a causal relationship between iNO use and worse outcomes.

Consistent with previous studies, increasing PPHN severity was associated with fewer VFDs, longer ventilation, and higher mortality [27]. The overall mortality rate (15%) and duration of mechanical ventilation in our study were comparable to those reported in previous studies [28].

Although data on VFDs in infants with PPHN remain limited, their interpretation differs from that of other outcomes such as mortality, duration of mechanical ventilation days, and NICU length of stay. Because VFDs assign a value of zero to both early death and prolonged ventilation beyond 28 days, they may mask important differences in clinical courses, even when patient outcomes differ. Therefore, VFDs should be interpreted alongside other neonatal outcomes to provide a more comprehensive assessment of clinical status and treatment response.

This study has several limitations. It was a single-center retrospective study conducted over an 11-year period. During this long span, clinical practices evolved substantially, which introduces potential temporal confounding and treatment-era bias that could not be analytically adjusted for in our statistical models. Furthermore, there is an inherent risk of confounding by indication; critical interventions such as iNO therapy, the use of multiple inotropic agents, and ECMO candidacy strongly reflect baseline disease severity and multisystem failure rather than independent causal determinants of poorer outcomes.

Additionally, certain etiologic subgroups were extremely small, particularly birth asphyxia (*n* = 3) and other causes (*n* = 11). Consequently, statistical comparisons across these etiologies suffer from limited statistical power and an increased risk of failing to detect true differences between groups; thus, these subgroup findings should be interpreted with caution and viewed as exploratory. In addition, long-term neurodevelopmental and respiratory outcomes beyond hospital discharge were not assessed. Furthermore, our findings reflect practices in a tertiary care setting with access to advanced respiratory support, which may limit generalizability to community or non-tertiary centers. Methodologically, treating VFDs as a time-to-event survival endpoint possesses inherent limitations due to its composite nature. Because VFDs assign a score of zero to both deceased patients and those requiring prolonged ventilation beyond 28 days, standard survival models inherently face the challenges of zero-inflation and competing risks. To mitigate this concern and ensure the robustness of our data, we conducted a sensitivity analysis restricted to 28-day survivors who were successfully extubated. This secondary analysis demonstrated a natural, non-zero-inflated probability curve, confirming that our core clinical findings regarding the factors associated with ventilation duration remain stable and are not artifactually driven by mathematical distortions from the mortality or prolonged ventilation cohorts. There is also a potential risk of reverse causation bias among the identified factors in our multivariable analysis. Specifically, VAP is strongly linked to the duration of mechanical ventilation, meaning that prolonged ventilation may have preceded and predisposed infants to VAP, rather than VAP acting purely as an independent antecedent predictor of lower VFDs. Consequently, these findings warrant validation in larger, multi-center prospective studies utilizing advanced statistical approaches such as competing risk analysis.

The strengths of this study include highlighting the clinical utility of VFDs as a composite outcome integrating survival and ventilator dependency, thereby providing a more comprehensive assessment of short-term outcomes in PPHN. Echocardiographic confirmation was obtained in more than 95% of cases, enhancing diagnostic accuracy. In addition, both initial and maximum OI were analyzed, capturing disease severity across early and critical phases. Finally, these findings support the potential role of VFDs as an outcome measure in future clinical studies and trials in PPHN, particularly when both survival and respiratory morbidity are of interest.

## 5. Conclusions

A maximum OI > 40, the use of more than two inotropic agents, AKI, and VAP were significantly associated with fewer VFDs in neonates with PPHN. When evaluated by etiology, infants with PPHN secondary to RDS had the fewest VFDs. Given our retrospective single-center design, these findings are associative and should be considered hypothesis-generating, reflecting overall clinical severity rather than definitive causal determinants. Nevertheless, VFDs effectively capture both mortality and respiratory morbidity. Consequently, VFDs offer a comprehensive framework to evaluate clinical outcomes, supporting their potential utility as a clinically meaningful outcome measure in future PPHN studies and clinical trials, particularly when both survival and respiratory morbidity are of interest. Future prospective, multi-center studies are required to validate these findings.

## Figures and Tables

**Table 1:** Baseline characteristics and outcomes of neonates with PPHN, stratified by severity: mild-to-moderate (OI ≤ 25) versus severe-to-critical (OI > 25).

Characteristic	Total (*n* = 175)	OI ≤ 25 (*n* = 115)	OI > 25 (*n* = 60)	*p*-Value
Gestation age, weeks *	37 (35–38)	37 (36–39)	36 (34–38)	0.006
Birth weight, g **	2794 ± 795	2909 ± 719	2574 ± 889	0.008
Cesarean section, *n* (%)	150 (85.7)	97 (84.3)	53 (88.3)	0.626
Cause of PPHN, n (%)				0.115
MAS	45 (25.7)	30 (26.1)	15 (25)	
TTN	50 (28.6)	39 (33.9)	11 (18.3)	
Pneumonia/sepsis	35 (20)	20 (17.4)	15 (25)	
RDS	31 (17.7)	18 (15.7)	13 (21.7)	
Birth asphyxia	3 (1.7)	3 (2.6)	0 (0)	
Other	11 (6.3)	5 (4.3)	6 (10)	
Echocardiography, *n* (%)	172 (98.3)	112 (97.4)	60 (100)	0.552
Pneumothorax, *n* (%)	30 (17.1)	13 (11.3)	17 (28.3)	0.009
Ventilator-free days, d *	20 (9–23)	22 (16.5–24)	8.5 (0–19)	<0.001
Duration of mechanical ventilation, d *	6 (4–11)	5 (4–9.5)	7 (5–15.5)	0.029
VAP, *n* (%)	12 (6.9)	8 (7)	4 (6.7)	0.99
Acute kidney injury, *n* (%)	47 (26.9)	29 (25.2)	18 (30)	0.619
BPD, *n* (%)	15 (8.6)	6 (5.2)	9 (15)	0.056
Length of hospital stay, d *	15 (10–23)	15 (10–21.5)	16 (6–28)	0.868
Death, *n* (%)	27 (15.4)	8 (7)	19 (31.7)	<0.001

* Median (IQR), ** mean ± standard deviation (SD). BPD, bronchopulmonary dysplasia; MAS, meconium aspiration syndrome; PPHN, persistent pulmonary hypertension of the newborn; RDS, respiratory distress syndrome; TTN, transient tachypnea of the newborn; VAP, ventilator-associated pneumonia.

**Table 2:** Factors associated with ventilator-free days in neonates with PPHN.

Variable	Univariate		Multivariate	
	HR (95% CI)	*p*-Value	Adjusted HR (95% CI)	*p*-Value
BW < 2500 g	1.47 (1.05–2.04)	0.027	−	−
GA < 37 weeks	1.61 (1.18–2.2)	0.003	−	−
5 min Apgar score < 5	1.86 (1.15–3.01)	0.019	−	−
RDS	1.53 (1.03–2.26)	0.043	−	−
TTN	0.79 (0.57–1.1)	0.151	−	−
Initial OI > 25	2.65 (1.91–3.66)	<0.001	−	−
Maximum OI > 40	3.43 (2.48–4.75)	<0.001	3.5 (2.49–4.9)	<0.001
Inotropic drug > 2 agents	2.43 (1.62–3.63)	<0.001	2.27 (1.49–3.45)	<0.001
Acute kidney injury	1.59 (1.14–2.23)	0.009	1.54 (1.1–2.17)	0.016
VAP	3.97 (2.12–7.43)	<0.001	3.42 (1.8–6.48)	<0.001
Pneumothorax	1.86 (1.24–2.79)	0.005	−	−

BW, birth weight; CI, confidence interval; GA, gestational age; HR, hazard ratio; RDS, respiratory distress syndrome; TTN, transient tachypnea of the newborn; OI, oxygenation index; VAP, ventilator-associated pneumonia.

**Table 3:** Neonatal outcomes according to the underlying causes of PPHN.

Outcome	RDS (*n* = 31)	TTN (*n* = 50)	MAS (*n* = 45)	Pneumonia /Sepsis (*n* = 35)	Birth Asphyxia (*n* = 3)	Other (*n* = 11)	*p*-Value
VFDs, d *	16 (0–22)	22 (15–24)	22 (11–24)	17 (7–21)	17 (8.5–20.5)	21 (18–25)	0.023
Duration of mechanical ventilation, d *	6 (3–8)	5(4–9)	5 (4–12)	9 (5.5–18)	11 (7.5–40.5)	5 (2.5–7.5)	0.021
Length of stay, d *	13 (5.5–25)	14 (10–19)	15 (10–24)	18 (14–28)	25 (22.5–60)	11 (9–16.5)	0.027
Death, n (%)	12 (38.7)	3 (6)	6 (13.3)	4 (11.4)	0 (0)	2 (18.2)	0.007

* presented as median (IQR). MAS, meconium aspiration syndrome; PPHN, persistent pulmonary hypertension of the newborn; RDS, respiratory distress syndrome; TTN, transient tachypnea of the newborn; VFD, ventilator-free days.

## Data Availability

The raw data supporting the conclusions of this study will be made available by the authors on request. The data are not publicly available due to privacy.

## Supplementary Materials

The following supporting information can be downloaded at: https://www.mdpi.com/article/10.3390/jcm15114377/s1, Figure S1: Sensitivity analysis of ventilator-free days (VFDs) cumulative probability curve, strictly excluding patients who died within 28 days (*n* = 142). This curve demonstrates the true time-to-extubation distribution among survivors, resolving the zero-inflation effect observed in the primary analysis.
