# Supplementary material for: Clinical Factors Associated with Ventilator-Free Days in Newborns with Persistent Pulmonary Hypertension of the Newborn: A Retrospective Cohort Study in Thailand
Source: J Clin Med. 2026 Jun 5;15(11):4377. doi: 10.3390/jcm15114377 (PMC13257450; doi:10.3390/jcm15114377)
Supplement: Supplementary file 1 [file jcm-15-04377-s001.zip › jcm-4316405-supplementary.pdf]

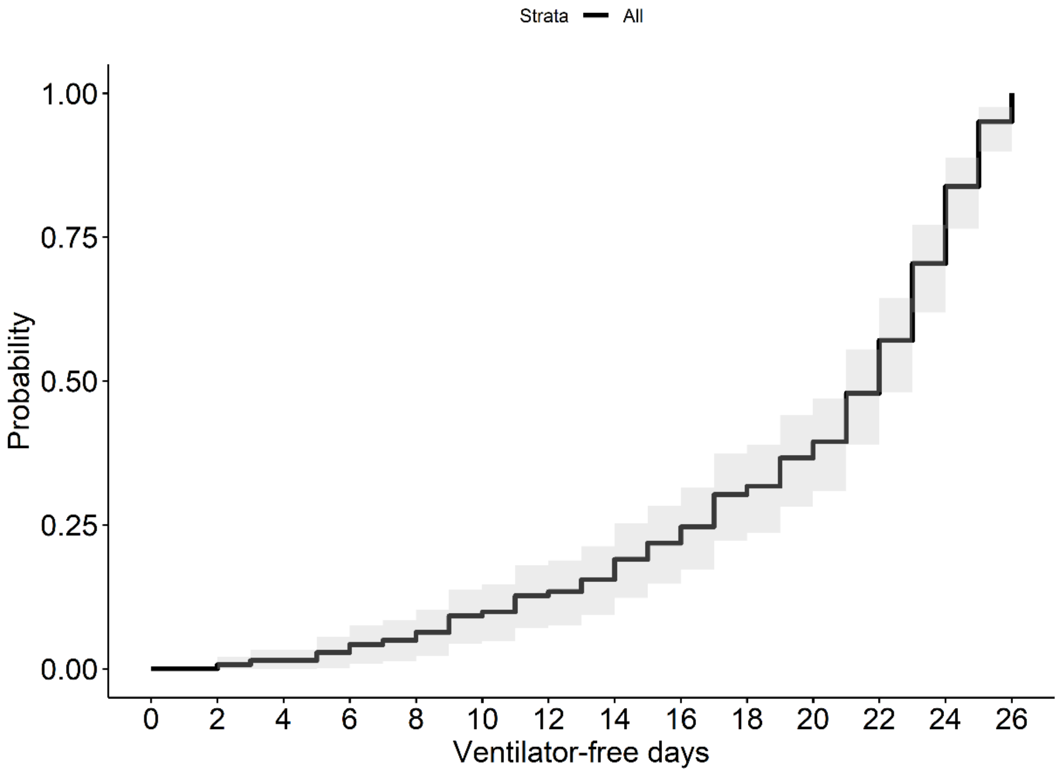

Supplementary Figure S1: Sensitivity analysis of ventilator-free days (VFDs) cumulative probability curve, strictly excluding patients who died within 28 days (N = 142). This curve demonstrates the true time-to-extubation distribution among survivors, resolving the zero-inflation effect observed in the primary analysis.
